# A novel ceRNA-immunoregulatory axis based on immune cell infiltration in ulcerative colitis-associated colorectal carcinoma by integrated weighted gene co-expression network analysis

**DOI:** 10.1186/s12876-022-02252-7

**Published:** 2022-04-15

**Authors:** Shi Yin, Xianzhe Li, Zhizhong Xiong, Minghao Xie, Longyang Jin, Huaxian Chen, Chaobin Mao, Fengxiang Zhang, Lei Lian

**Affiliations:** 1 Department of Gastrointestinal Surgery, The Sixth Affiliated Hospital of Sun Yat-sen University, Guangzhou, China; 2 Guangdong Institute of Gastroenterology, Guangdong Provincial Key Laboratory of Colorectal and Pelvic Floor Diseases, The Sixth Affiliated Hospital of Sun Yat-sen University, Guangzhou, China

**Keywords:** NEAT1, miR-1-3p, IL6ST, Immune microenvironment, Ulcerative colitis-associated colorectal carcinoma

## Abstract

**Background:**

Patients with ulcerative colitis are at an increased risk of developing colorectal cancer with a prolonged disease course. Many studies have shown that alterations in the immune microenvironment play a key role in ulcerative colitis-associated colorectal cancer. Additionally, competing endogenous RNAs have important functions in immunoregulation, affecting inflammation and tumorigenesis. However, the complexity and behavioral characteristics of the competing endogenous RNA immunoregulatory network in ulcerative colitis-associated colorectal cancer remain unclear. We constructed a competing endogenous RNA immunoregulatory network to discover and validate a novel competing endogenous RNA immunoregulatory axis to provide insight into ulcerative colitis-associated colorectal cancer progression.

**Methods:**

The competing endogenous RNA immunoregulatory network was constructed using differential expression analysis, weighted gene co-expression network analysis, and immune-related genes. Cmap was used to identify small-molecule drugs with therapeutic potential in ulcerative colitis-associated colorectal cancer. The ulcerative colitis-associated colorectal cancer-related pathways were identified by gene set variation and enrichment analysis. CIBERSORT, single-sample Gene Set Enrichment Analysis, and xCell were used to evaluate the infiltration of immune cells and screen hub immunocytes. The competing endogenous RNA immunoregulatory axis was identified by correlation analysis.

**Results:**

We identified 130 hub immune genes and constructed a competing endogenous RNA immunoregulatory network consisting of 56 long non-coding RNAs, four microRNAs, and six targeted hub immune genes. Four small-molecule drugs exerted potential therapeutic effects by reversing the expression of hub immune genes. Pathway analysis showed that the NF-κB pathway was significantly enriched. Neutrophils were identified as hub immunocytes, and IL6ST was significantly positively correlated with the neutrophil count. In addition, NEAT1 may serve as a competing endogenous RNA to sponge miR-1-3p and promote IL6ST expression.

**Conclusions:**

The competing endogenous RNA immunoregulatory axis may regulate neutrophil infiltration, affecting the occurrence of ulcerative colitis-associated colorectal cancer.

**Supplementary Information:**

The online version contains supplementary material available at 10.1186/s12876-022-02252-7.

## Background

Ulcerative colitis (UC) is a chronic, recurrent, and intestinal inflammatory disease; however, its pathogenesis remains unclear. It is generally considered that the intestinal mucosal immune system of patients with UC produces abnormal amplification of the immune response to intestinal microbial antigens in a specific environment, which can cause intestinal mucosal inflammatory damage [1]. The imbalance between inflammation and mucosal immunity is an important feature of colorectal carcinogenesis [2]. Therefore, patients with UC have an increased risk of UC-associated colorectal cancer (CAC).

Although an increasing number of studies have investigated coding gene-related biomarkers in CAC, protein-coding genes only account for 2% of the human genome. The competitive regulatory crosstalk of different molecular species, especially the competition between protein-coding and non-coding RNA transcripts, is a key link in the occurrence of disease [3, 4]. MicroRNAs (miRNAs), as an abundant class of small, non-coding, single-stranded oligoribonucleotides, act as regulators in various cellular processes and function as sequence-specific silencers of target gene transcription after binding, thereby affecting the expression levels of more than half of all protein-coding genes [5, 6]. Therefore, Salmena et al. [7] proposed the competing endogenous RNA (ceRNA) hypothesis, which posits that most RNA molecules act in a “many-to-many” manner. As each miRNA molecule may potentially target miRNA response elements on multiple messenger RNA (mRNA) molecules, each mRNA molecule can be targeted by multiple miRNA molecules [8]. This suggests that miRNA molecules have a pivotal role in competitively regulating crosstalk and lead to gene silencing by binding to mRNAs, and that ceRNA regulates gene expression by competitively binding to miRNAs.

Numerous ceRNA studies in different diseases (ie tumors, inflammatory bowel diseases, liver fibrosis, and cardiovascular diseases) have suggested that ceRNA can influence dysregulation of the immune microenvironment by regulating the interaction of different types of RNAs, thereby promoting the occurrence and development of diseases [9–15]. However, the specific immunoregulatory mechanisms in the CAC process remain unclear. We hypothesized that the ceRNA regulatory axis can alter the immune microenvironment during the development of CAC.

To verify this hypothesis, we integrated immune-related genes (IRGs), weighted gene co-expression network analysis (WGCNA), and differential expression analysis results, and then constructed a ceRNA immunoregulatory network based on the principle of reverse prediction. Subsequently, small-molecule medicines with possible applications in therapy for CAC were investigated. Pathway enrichment analysis and immune cell infiltration analysis were performed to identify the key pathways and immune cells. Correlation analysis was conducted to identify the key ceRNA regulatory axis from the network. This study provides a foundation for improving the understanding of the pathophysiological processes of CAC and insights into the variations in the immunological microenvironment of the disease.

## Methods

### Data acquisition and preprocessing

**Fig. 1:**
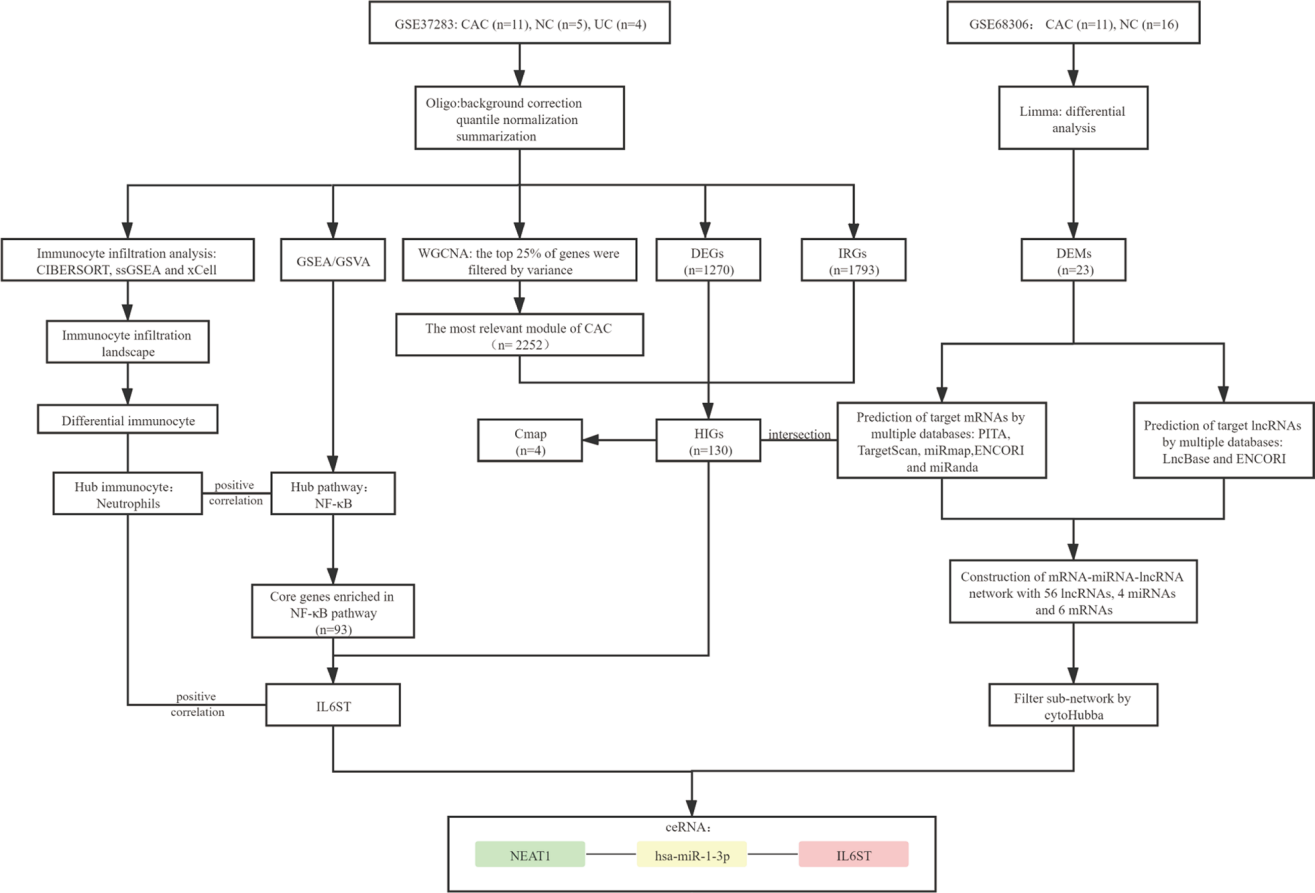
Research and design flow chart. *UC* ulcerative colitis, *NC* normal control, *CAC* ulcerative colitis-associated colorectal cancer, *DEGs* differentially expressed genes, *IRGs* immune-related genes, *DEMs* differentially expressed micro RNAs, *HIGs* hub immune genes

The research and design flowchart is shown in Fig. 1. Gene expression profiles of datasets GSE37283 [16] and GSE68306 [17] were downloaded from the National Center for Biotechnology Information (NCBI).

Gene Expression Omnibus (GEO) database (https://www.ncbi.nlm.nih.gov/geo/). The GSE37283 dataset contains mRNA microarray raw data from 20 colonic mucosal samples (five normal control [NC] samples, four UC samples, and 11 UC samples harboring remote neoplasia). The GSE68306 dataset contains miRNA normalized expression matrix data of 11 UC-associated neoplastic samples and 16 NC samples. Raw CEL data (data storage format) were parsed using the ‘affy’ package in R [18]. The robust multiarray average (RMA) algorithm background correction and quantile normalization were performed on gene expression profiles with the ‘oligo’ package in R [19]. The annotation package of the microarray platform GPL13158 was used to convert the probe IDs into gene symbols. Chip quality was assessed using the ‘arrayQualityMetrics’ package in R [20]. Moreover, 1793 IRGs were downloaded from the Immunology Database and Analysis Portal database (https://immport.niaid.nih.gov), which covers 17 immune categories [21].

### Construction of weighted gene co-expression gene network

The ‘WGCNA’ package in R was used to analyze the co-expression network of the top 25% gene variants in the GSE37283 dataset [22]. The adjacency matrix was constructed by calculating Pearson’s correlation coefficient. Subsequently, a scale-free co-expression network based on the soft threshold parameter β was established. The adjacency matrix was converted to a topological overlap matrix (TOM) by comparing the weighted correlation of the two nodes with the others. Hierarchical clustering of dissimilarity TOM (dissTOM = 1 − TOM) rendered similar gene expression levels in the same gene module. Next, the dynamic cut tree algorithm was used to further partition the modules. Additionally, module eigengenes (MEs) and gene significance (GS) were used to identify modules associated with CAC. MEs represent the principal component of all gene expression levels in individual modules. GS was considered as the mediated *p*-value for each gene, representing the correlation between gene expression and CAC.

### Differential expression analysis and identification of hub immune genes

We analyzed the differentially expressed mRNAs and miRNAs between CAC and NC samples using the classical Bayesian methodology with the ‘limma’ package in R [23]. In GSE37283, the cut-off criteria were set at *p* < 0.05 and |log2fold-change (FC)|> 1. In GSE68306, we adjusted the threshold of differential expression to *p* < 0.05 and |log2FC|> 0.5, allowing the results to be optimized. The differentially expressed mRNAs, IRGs, and CAC-related module genes in WGCNA were intersected to obtain the differentially expressed hub immune genes (HIGs), which were visualized in a Venn diagram [24].

### Small-molecule drug prediction

The Cmap database (http://www.broadinstitute.org) includes and collates 6100 gene expression profiles from 7056 microarray datasets, covering 1309 Food and Drug Administration-approved small-molecule drugs [25]. The database is commonly used to predict potential drugs for treating diseases and repurposing existing drugs. Uploading the HIGs into the Cmap database resulted in connectivity scores for the corresponding small-molecule drugs with values between + 1 and − 1. A positive connectivity score indicated that the small-molecule drug induced gene expression, whereas a negative connectivity score suggested that specific drugs could reverse gene expression patterns. Therefore, the screening criteria for potential therapeutic drugs were enrichment < −0.7, and *p* < 0.01. The molecular structures of potential therapeutic drugs were displayed using the PubChem database (https://pubchem.ncbi.nlm.nih.gov/) [26].

### Gene set enrichment analysis

Gene set enrichment analysis (GSEA) and gene set variation analysis (GSVA) are two types of gene enrichment methods [27, 28], the former estimates the enrichment of pathways in phenotypic genes according to the background gene set, whereas the latter transforms the gene expression matrix into a gene set enrichment matrix using an unsupervised method. We downloaded the hallmark gene set from the Molecular Signatures Database (MSigDB; https://www.gsea-msigdb.org/gsea/msigdb/index.jsp) for use as reference [29]. The ‘clusterpProfiler’ package in R was used for GSEA and visualization [30]. The *p* and *q* values were obtained by simulating permutations of 1000 random genomes, and *p* < 0.05 and *q* < 0.05 were used as screening criteria. For GSVA, we used the ‘gsva’ and ‘limma’ packages in R to screen pathways with significant differences (*p* < 0.05 and |log2FC|> 0.5), and the results were displayed with the ‘pheatmap’ package [23, 28].

### Immune cell infiltration analysis

Many algorithms have been developed to evaluate the infiltration level of immune cell populations, among which CIBERSORT, single-sample GSEA (ssGSEA), and xCell are the most widely used research methods [31, 32]. For CIBERSORT, we estimated the proportion of 22 immune cell populations in the samples using the support vector regression machine learning method with the ‘cibersort’ package in R [33]. For ssGSEA, we compared 28 types of immune cell characteristic gene sets and converted the gene expression values of samples into the enrichment fraction to obtain the relative abundance of immune cells in samples using the ‘gsva’ package in R [34]. For xCell, we performed a cell type enrichment analysis from the gene expression data of 64 immune and stromal cell types using the ‘xcell’ package in R. We subsequently identified immune cell types that were significantly different in CAC and normal tissue using the Wilcoxon test.

### ceRNA network construction

ENCORI (http://starbase.sysu.edu.cn/) is a comprehensive database that provides experimentally supported miRNA-mRNA and miRNA-long non-coding RNA (lncRNA) interaction networks [35]. In addition, it integrates information from multiple miRNA-mRNA prediction databases, including PITA, miRmap, miRanda, and TargetScan. To ensure the accuracy of predicting target genes, only those that existed in the four above-mentioned databases were included in the regulatory network. Subsequently, differentially expressed mRNAs (DEmRNAs) were intersected with predicted mRNAs to explore differentially expressed miRNA-mRNA regulatory relationship pairs in CAC. Next, the roles of differentially expressed miRNAs (DEmiRNAs) and lncRNAs were predicted using the ENCORI and LncBase databases [36]. The network was constructed by screening out the lncRNA-miRNA-mRNA regulatory axis that was regulated by the same DEmiRNAs and visualized in Cytoscape 3.8.2 [37]. To further identify the key targets in the process of CAC, the Cytoscape plugin ‘Cytohubba’ was used to perform non-weighted parameter analysis of node connectivity in the network [38]. Finally, the subnetwork was screened according to the connectivity score. Based on the ceRNA hypothesis, lncRNA plays a sponge adsorption role in the cytoplasm and is positively correlated with mRNA expression levels. Therefore, we used LNCipedia (https://Incipedia.org/) to obtain the lncRNA sequence and determine its cellular location using the lncLocator database (http://www.csbio.sjtu.edu.cn/bioinf/lncLocator/) [39, 40]. Co-expression analysis of lncRNA and mRNA was performed based on The Cancer Genome Atlas colorectal cancer data compiled by the ENCORI database. Additionally, CentroidFold (http://rtools.cbrc.jp/centroidfold/) was used to predict the secondary structure of the miRNA precursor stem-loop [41]. Statistical significance was set at *p* < 0.05.

### Statistical analysis

Data analysis was performed with R 4.0.3. The Wilcoxon test was used to compare the types of immune cells in the CAC and NC groups. Spearman’s correlation analysis was used to assess the correlation between the two groups. The chi-square test was used for categorical variables. Statistical significance was set at *p* < 0.05.

## Results

### Chip quality analysis

**Fig. 2:**
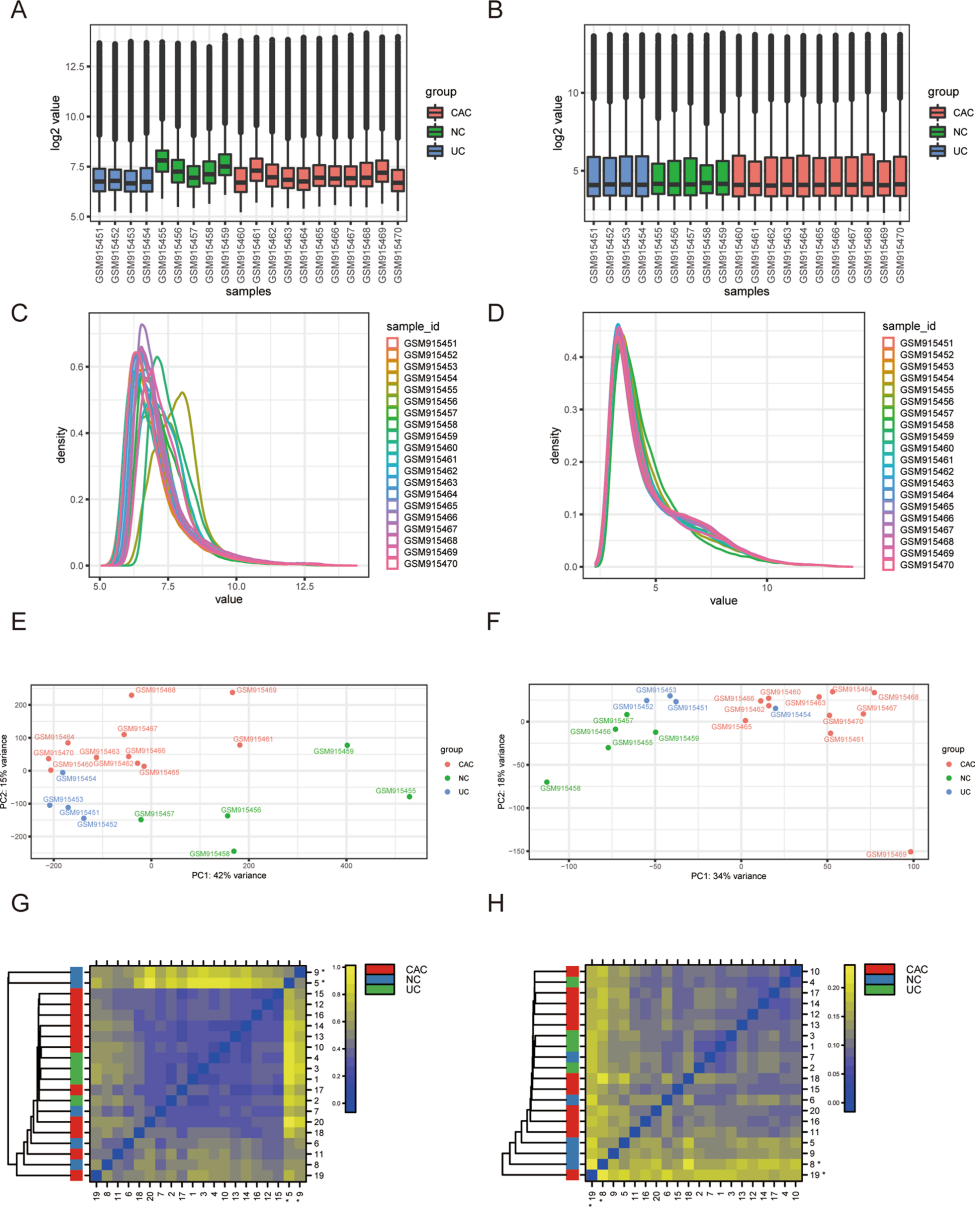
Chip data correction and evaluation. Boxplots show gene expression levels before (**A**) and after (**B**) correction. Density plots show estimates of data density before (**C**) and after (**D**) normalization. PCA is a dimension reduction and visualization technique used to project the multivariate data vector of each array into a two-dimensional plot, and thus, the spatial arrangement at the midpoint of the plot reflects similarity between the overall data. PCA before processing (**E**) and shows after processing (**F**). Heatmaps before correction (**G**) and after correction (**H**) show the array aggregation caused by expected biological or unexpected experimental factors (batch effects). Outliers are denoted by *. *PCA* principal component analysis

To evaluate the quality of the chip and increase the reliability of the results, we compared the differences in raw data obtained from the GSE37283 dataset of the GEO database before and after correction using boxplots, smoothed histograms, principal component analysis (PCA), and array clustering heatmaps. Boxplots detected outliers by calculating the distribution of each array using pooled data, whereas the smoothed histograms estimated the density of data to indicate the chip quality-related phenomenon. We found many raw data outliers and large differences in density curves among samples that required normalization to eliminate the effect of systematic errors (Fig. 2A–D). Therefore, PCA and array clustering heatmaps were used to describe batch effects among samples (Fig. 2E–G). Although the influence of the batch effect was markedly reduced, restoring the actual biological characteristics of the chip, there were still two abnormal samples (GSM915458 and GSM915469) in the corrected data (Table 1). Overall, these results showed that two outliers must be removed to ensure the reliability of subsequent analysis based on the chip quality control method.

**Table 1:** Overview of chip quality control

Array	Samplenames	*1	*2	*3	Group
1	GSM915451	–	–	–	UC
2	GSM915452	–	–	–	UC
3	GSM915453	–	–	–	UC
4	GSM915454	–	–	–	UC
5	GSM915455	–	–	–	NC
6	GSM915456	–	–	–	NC
7	GSM915457	–	–	–	NC
8	GSM915458	–	–	√	NC
9	GSM915459	–	–	–	NC
10	GSM915460	–	–	–	CAC
11	GSM915461	–	–	–	CAC
12	GSM915462	–	–	–	CAC
13	GSM915463	–	–	–	CAC
14	GSM915464	–	–	–	CAC
15	GSM915465	–	–	–	CAC
16	GSM915466	–	–	–	CAC
17	GSM915467	–	–	–	CAC
18	GSM915468	–	–	–	CAC
19	GSM915469	–	–	√	CAC
20	GSM915470	–	–	–	CAC

Columns named as *1, *2, and *3 indicate calls from different outlier detection methods: (1) outlier detection by boxplots; (2) outlier detection by distances between arrays (density plots); and (3) batch effect detection using principal component analysis plots and array clustering heatmaps. “√” indicates the presence of an outlier; “–” indicates the absence of an outlier

*UC* ulcerative colitis, *NC* normal control, *CAC* ulcerative colitis-associated colorectal cancer

### Weighted co-expression network construction and identification of key modules

**Fig. 3:**
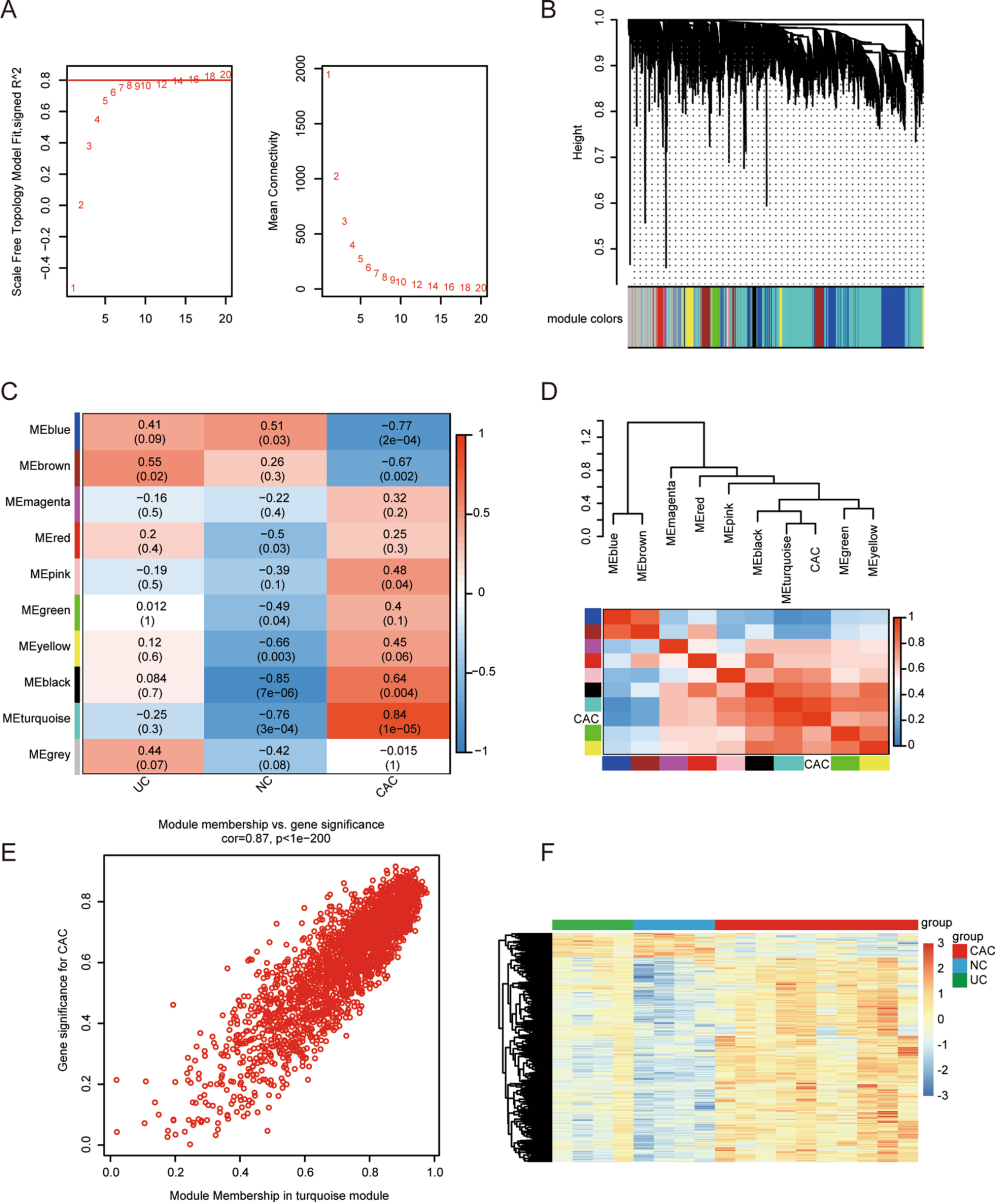
Construction of weighted co-expression network and module analysis. **A** Scale-free index under various soft threshold power (β) and average connectivity analysis. **B** Cluster dendrogram of co-expression network module. Each color represents one specific co-expression module. **C** Heatmap of the correlation between the module eigengenes and clinical traits. Each row represents a color module and every column a clinical trait (UC, NC, and CAC). Each cell contains the corresponding correlation and p-value. Statistical significance was set at *p* < 0.05. **D** Dendrogram (top) and heatmap (bottom) display the strength of correlations between CAC and other modules. Red represents a higher positive adjacency and blue a lower adjacency. **E** Correlation between module membership of turquoise module and gene significance with CAC. **F** Heatmap with clusters of DEGs in turquoise module among the three different groups. *UC* ulcerative colitis, *NC* normal control, *CAC* ulcerative colitis-associated colorectal cancer, *DEGs* differentially expressed genes

After the analysis of variance (ANOVA), the top 25% of genes (4980 genes) from the 18 samples screened for sequencing were subjected to WGCNA to identify genes highly associated with CAC. To define the adjacency matrix, we introduced weight parameters when soft-thresholded β = 16 (scale-free R^2^ > 0.8) and scale-free networks were constructed (Fig. 3A). The dynamic tree-cutting algorithm classified the 4980 genes into 10 co-expression modules (Fig. 3B; Additional file 1: Table S1). By calculating the correlation of each module with the corresponding clinical features, we found that genes in the MEturquoise module were most strongly associated with CAC (r = 0.84, *p* < 0.01) and were identified as key modules in the CAC process (Fig. 3C, D). The MEturquoise module contained 2264 genes. Further statistical tests on the gene significance (GS) of MEturquoise module membership (MM) and CAC showed that 2264 genes contributed significantly to the membership of the MEturquoise module and CAC (r = 0.87, *p* < 0.01) (Fig. 3E). Moreover, hierarchical clustering analysis of gene expression levels in the MEturquoise module revealed that gene expression in the CAC group differed from that in the UC and NC groups (Fig. 3F). Therefore, these genes co-expressed with CAC require further study.

### GSVA and GSEA reveal CAC-related pathways

**Fig. 4:**
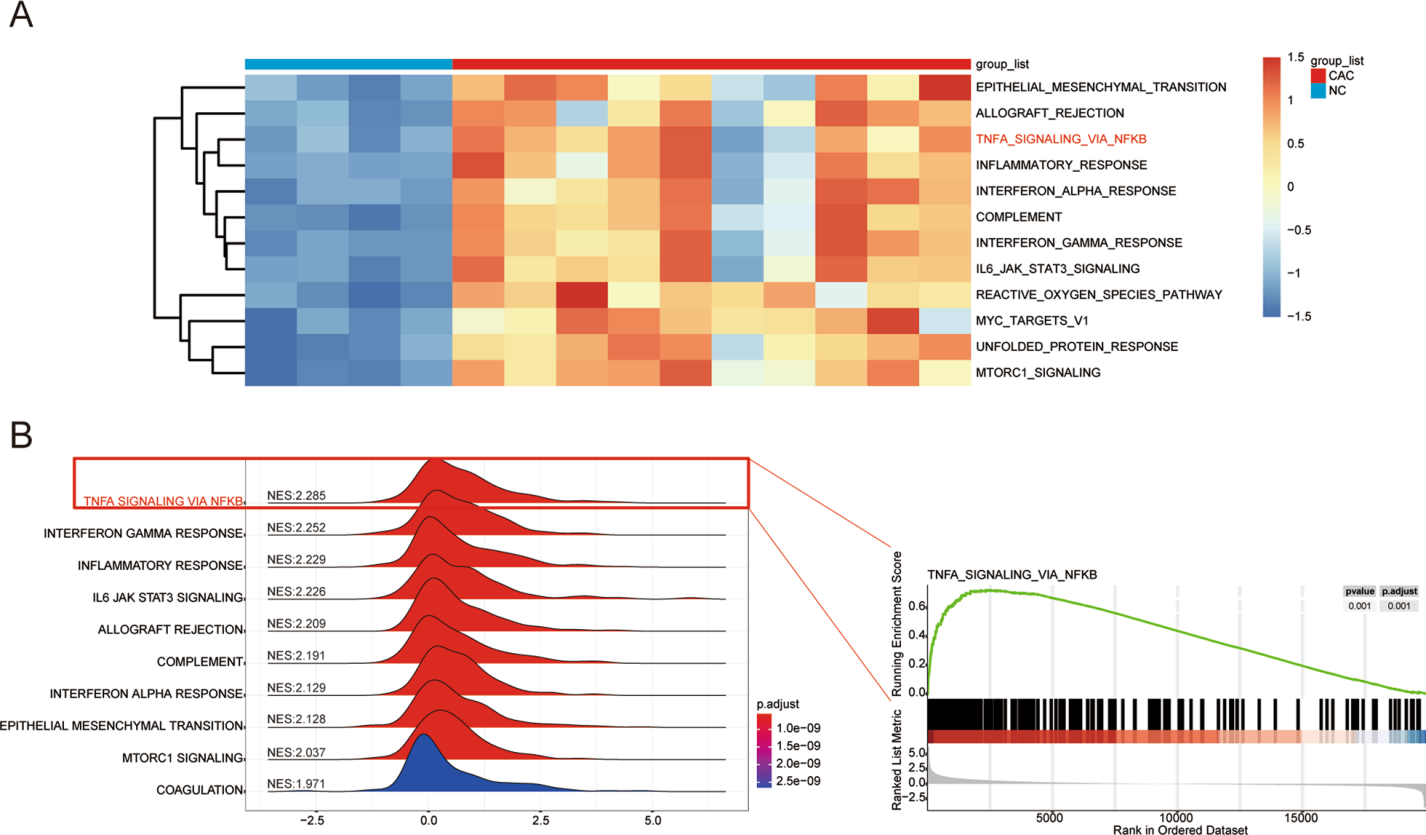
CAC-related pathways analysis. **A** Heatmap showing significantly different pathways in GSVA; *p* < 0.05 and |log2FC|> 0.5. **B** Ridge plots with normalized enrichment score displaying the 10 most enriched pathways of CAC in GSEA. *CAC* ulcerative colitis-associated colorectal cancer, *GSVA* gene set variation analysis, *NES* normalized enrichment scores, *GSEA* gene set enrichment analysis

Using *p* < 0.05, and |log2FC|> 0.5, 12 pathways with significant changes were screened out (Fig. 4A; Table 2). According to the threshold value (*p* adjust < 0.05), we obtained the top 10 pathways with normalized enrichment scores from the results of GSEA. By combining the results of GSEA and GSVA, we found that TNFα signaling via the NF-κB pathway showed significant changes in CAC and had the highest degree of enrichment (normalize enrichment score: 2.285) (Fig. 4B).

**Table 2:** Differentially expressed pathways in gene set variation analysis

Pathway name	Type	Log2FC	p-value	Adj.p.val
INTERFERON_ALPHA_RESPONSE	Up	0.634121921	4.59E−05	0.000286711
INTERFERON_GAMMA_RESPONSE	Up	0.624598993	2.25E−05	0.000160714
IL6_JAK_STAT3_SIGNALING	Up	0.607187614	2.20E−05	0.000160714
UNFOLDED_PROTEIN_RESPONSE	Up	0.582015106	2.21E−06	6.29E−05
MTORC1_SIGNALING	Up	0.574645409	2.51E−06	6.29E−05
TNFA_SIGNALING_VIA_NFKB	Up	0.565526025	0.000174553	0.000809951
ALLOGRAFT_REJECTION	Up	0.555113783	8.06E−05	0.00044773
MYC_TARGETS_V1	Up	0.535347867	1.26E−05	0.000156916
REACTIVE_OXYGEN_SPECIES_PATHWAY	Up	0.517546923	8.42E−06	0.00014027
EPITHELIAL_MESENCHYMAL_TRANSITION	Up	0.508782095	0.000178189	0.000809951
INFLAMMATORY_RESPONSE	Up	0.505207341	0.000244903	0.000941935
COMPLEMENT	Up	0.502336993	1.98E−05	0.000160714

### Identification of CAC-related hub immune genes and small molecule therapeutic drug prediction

**Fig. 5:**
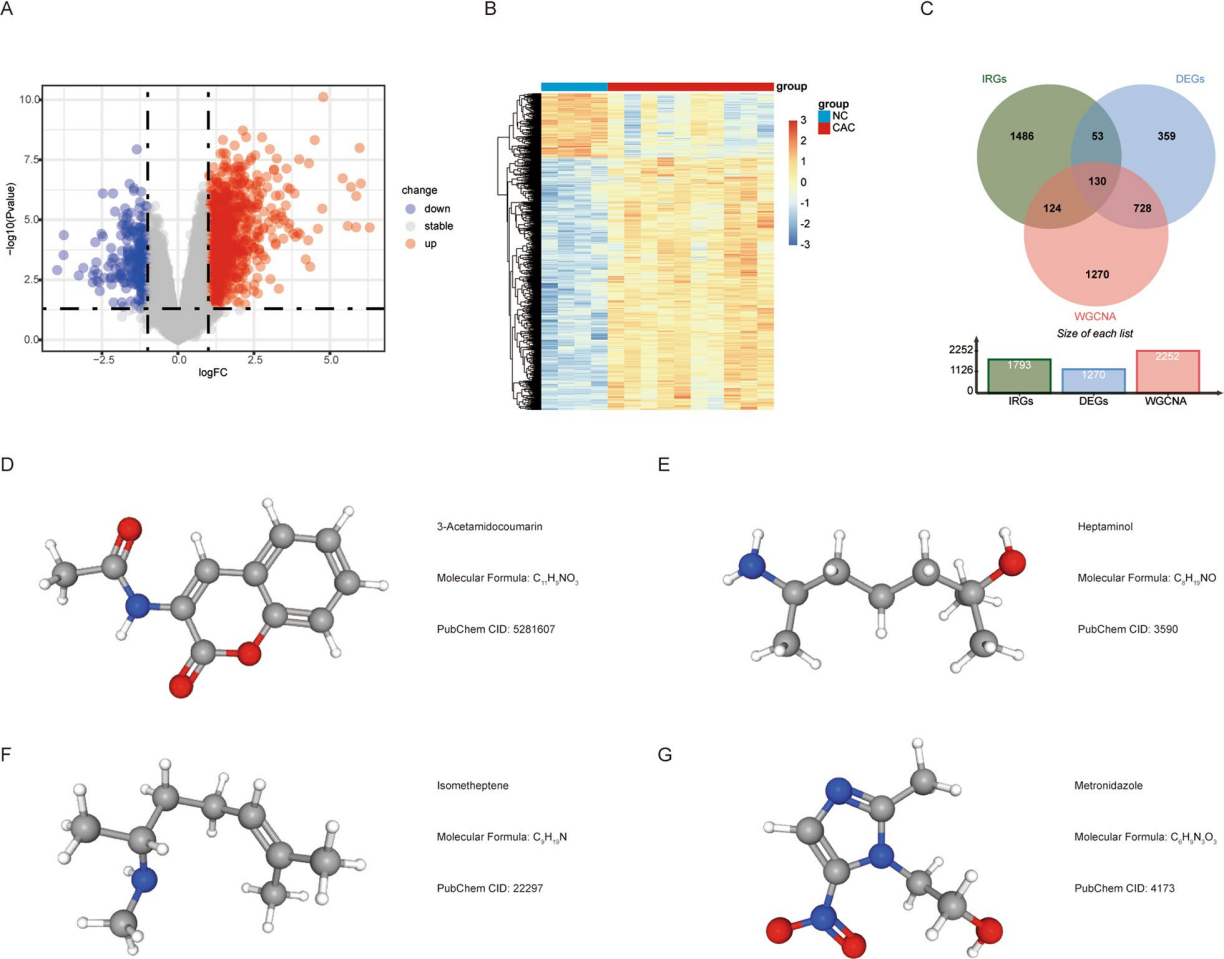
Identification of HIGs and small-molecule therapeutic drugs. Volcano plot (**A**) and heatmap (**B**) of DEGs between CAC and NC, *p* < 0.05, |log2FC|> 1. **C** Venn diagram displaying genes overlap in IRGs, GSEA and WGCNA results. **D**–**G** Prediction results of potential small-molecule drugs for treating CAC based on HIGs. *HIGs* hub immune genes, *DEGs* differentially expressed genes, *CAC* ulcerative colitis-associated colorectal cancer, *NC* normal control, *IRGs* immune-related genes, *GSEA* gene set enrichment analysis, *WGCNA* weighted gene co-expression network analysis

We performed differential expression analysis of the GSE37283 microarray expression matrix and filtered out 1271 DEGs, which met the criteria (*p* < 0.05, |log2FC|> 1) between the CAC and NC groups; of these genes, 1012 were upregulated and 258 were downregulated (Fig. 5A). Subsequent hierarchical clustering analysis of the 1270 DEGs revealed significantly different expression between the two groups (Fig. 5B). We then intersected 1793 immune genes downloaded from the Immport database, 2252 genes obtained from CAC modules in WGCNA, and DEGs to identify 130 HIGs (Fig. 5C; Additional file 1: Table S2). Based on the importance of HIGs in CAC, we explored agents that could reverse gene expression and showed therapeutic potential. Four drugs from the Cmap database met the criteria (enrichment < −0.7 and *p* < 0.01), and their three-dimensional molecular structures were displayed in the PubChem database (Fig. 5D–G; Table 3).

**Table 3:** Potential drugs with therapeutic potential for ulcerative colitis-associated colorectal cancer

Cmap name	Mean	N	Enrichment score	*p*	Specificity score	Percent non-null
Metronidazole	− 0.37	5	− 0.836	0.00032	0	80
3-Acetamidocoumarin	− 0.252	4	− 0.819	0.00203	0.039	50
Heptaminol	− 0.281	5	− 0.735	0.00272	0.0206	60
Isometheptene	− 0.23	4	− 0.799	0.00322	0.0138	50

Mean, average connectivity score; *N* number of instances

### Identification of differentially expressed miRNA and ceRNA network construction

**Fig. 6:**
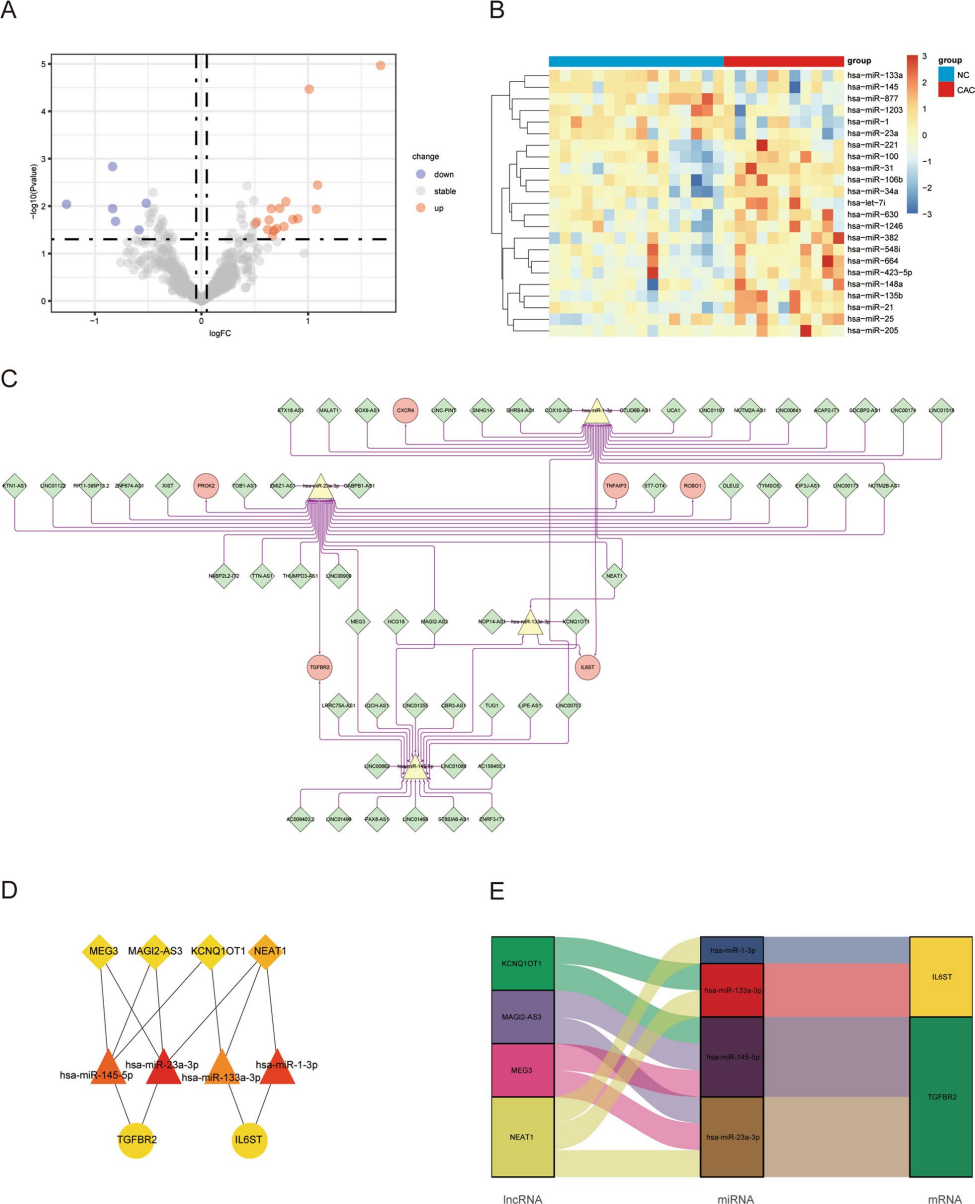
Construction of ceRNA immunoregulation network. Volcano plot (**A**) and heatmap (**B**) of DEMs between CAC and NC, *p* < 0.05, |log2FC|> 0.5. **C** Potential lncRNA-miRNA-mRNA immunoregulation network in CAC. The diamond represents the lncRNAs; triangle represents the miRNAs; and circle represents the mRNAs. Use Cytoscape's plugin Cytohubba to filter sub-networks (**D**), and visualize the regulatory relationships of sub-networks (**E**). *ceRNA* competing endogenous RNA, *DEMs* differentially expressed miRNAs, *CAC* ulcerative colitis-associated colorectal cancer, *NC* normal control, *miRNA* micro RNA, *mRNA* messenger RNA

We obtained 23 DEmiRNAs (17 upregulated and six downregulated) from the GSE68306 dataset using the same differential analysis method (Fig. 6A, B). Guided by the ceRNA hypothesis, we reversely predicted the mRNAs and lncRNAs downstream and upstream of the 23 miRNAs, respectively. Based on the ENCORI database, DEmiRNAs were matched with the potential mRNAs. Next, we matched the predicted target genes with the HIGs and identified eight pairs of miRNA-mRNAs, which contained four DEmiRNAs and six mRNAs. Next, based on the predicted lncRNAs upstream of the four DEmiRNAs, we used the intersection of the ENCORI and LncBase databases to identify 63 pairs of lncRNAs-miRNAs, including 56 lncRNAs. Finally, we integrated 56 lncRNAs, four miRNAs, and six targeted HIGs into a lncRNA-miRNA-mRNA network (Fig. 6C). Each node of the network was subjected to connectivity analysis using cytohubba, resulting in a subnetwork containing 10 nodes (Fig. 6D; Table 4). Overall, the ceRNA regulatory axis in the subnetwork may be closely related to the occurrence and development of CAC (Fig. 6E).

**Table 4:** Competing endogenous RNA immune regulatory subnetwork

Name	Type	Degree	MCC
hsa-miR-23a-3p	miRNA	25	25
hsa-miR-1-3p	miRNA	21	21
hsa-miR-145-5p	miRNA	20	20
hsa-miR-133a-3p	miRNA	5	5
NEAT1	lncRNA	3	3
IL6ST	mRNA	2	2
TGFBR2	mRNA	2	2
MAGI2-AS3	lncRNA	2	2
KCNQ1OT1	lncRNA	2	2
MEG3	lncRNA	2	2

*MCC* maximal clique centrality

### Immune landscape of CAC

**Fig. 7:**
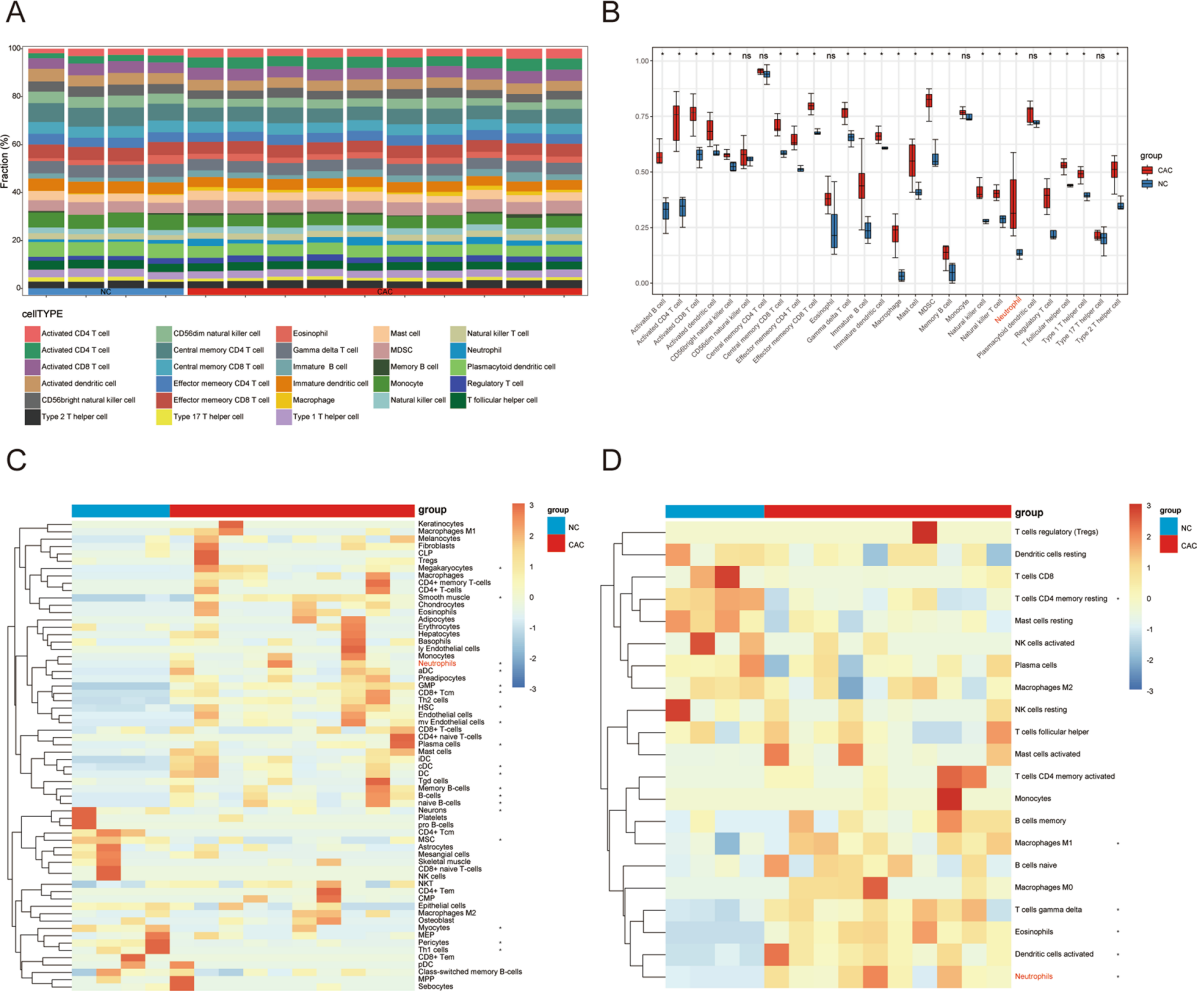
Immune cell infiltration analysis. **A** Percentage stacked bar chart shows the distribution of 28 immune cells in the CAC and NC group samples from the GSE37283 data set. **B** Results of ssGSEA showed differences in the abundance of immune cells between NC and CAC groups. Blue represents the NC group and red the CAC group. The heatmap demonstrates the statistics of immune cell infiltration results by the xCell (**C**) and CIBERSORT (**D**) algorithms. * indicates statistical significance at p < 0.05. *CAC* ulcerative colitis-associated colorectal cancer, *NC* normal control, *ssGSEA* single-sample gene set enrichment analysis

Previous studies demonstrated that inflammation or tumor initiation and progression are associated with the immune microenvironment and that immunocytes are key components in the microenvironment [42]. Therefore, exploration of the immune landscape in CAC is highly warranted. We first performed ssGSEA to quantify the relative abundance of immune cell infiltration in the CAC group compared to in the NC group (Fig. 7A). Wilcoxon analysis showed that 22 types of immune cells were significantly different between the two groups (*p* < 0.05) (Fig. 7B). Subsequently, CIBERSORT and xCell were used to evaluate immune cell infiltration and reduce bias before visualization in heatmaps (Fig. 7C, D). The most important observation from data comparison was that the neutrophils were the central immune cells, as they were the only cells showing the same trend in differential expression across the three algorithms.

### Identification and verification of a ceRNA-immunoregulation axis

**Fig. 8:**
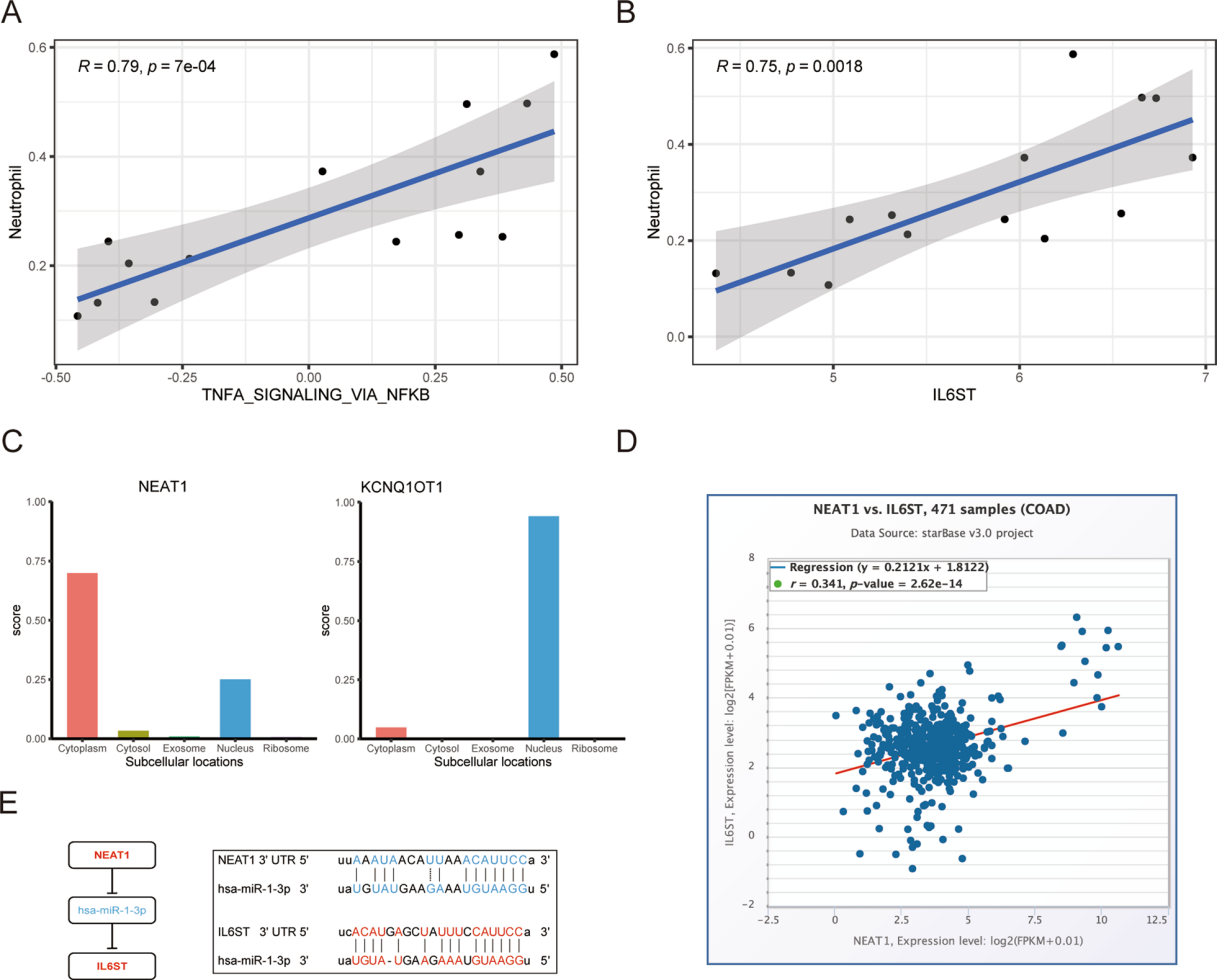
Identification of ceRNA-immunoregulatory axis. **A** Correlation analysis of the NF-κB pathway and neutrophils. **B** Correlation analysis of IL6ST and neutrophils. **C** Bioinformatics predict cellular localization of the lncRNAs NEAT1 and KCNQ1OT1. **D** Correlation plot of NEAT1 and IL6ST expression using the ENCORI database. **E** Schematic model of ceRNA; blue represents downregulation and red upregulation. Base pairing between miR-1-3p and the target sites of NEAT1 and IL6ST 3′ untranslated region as predicted using the database

Overall, the significant difference between the NF-κB pathway and neutrophils in CAC indirectly demonstrated that both factors play critical roles in CAC formation. Consequently, we carried out correlation analysis, which showed that the NF-κB pathway was significantly positively correlated with neutrophils (r = 0.79, *p* < 0.01) (Fig. 8A). We also intersected the core genes enriched in the NF-κB pathway with HIGs to obtain two key immune genes, IL6ST and TNFAIP3. Of these, IL6ST was significantly positively correlated with neutrophils (r = 0.75, *p* < 0.01) (Fig. 8B). To further analyze the regulatory mechanism of IL6ST at the transcriptional level in the ceRNA immunoregulatory network, we identified two upstream lncRNAs, NEAT1 and KCNQ1OT1 that regulate IL6ST. The mechanism of lncRNA is related to its localization in cells [43]. We predicted the subcellular localization of lncRNAs using the lnclocator algorithm. NEAT1 was mainly distributed in the cytoplasm, whereas KCNQ1OT1 in the nucleus (Fig. 8C). Using the ENCORI database, we verified that NEAT1 promotes the expression of the target mRNA IL6ST (Fig. 8D). We then predicted the precursor stem loop structures for the two target miRNAs of NEAT1 (Additional file 2: Fig. S1). Compared with miR-133a-3p, miR-1-3p had the highest connectivity score in the ceRNA network and a higher predictive score for interacting with NEAT1. These results indicate that NEAT1 absorbs miR-1-3p through sponge action to increase the expression of IL6ST (Fig. 8E).

## Discussion

UC is a chronic inflammatory disease of unknown etiology, and its cumulative incidence of progressing to CAC has markedly increased over the years [44]. Because of chronic inflammation caused by UC, various layers of the intestinal wall are infiltrated by immune cells, forming an immune microenvironment and participating in the induction of CAC through the production of cytokines and chemokines [45]. However, the underlying mechanisms remain unclear. Here, we explored the role of the ceRNA-immunoregulatory axis in the immune regulation of CAC at the transcriptional level.

We integrated three bioinformatics methods, including immune gene list, WGCNA, and differential gene expression analysis, to identify 130 HIGs. We found that metronidazole, 3-acetamidocoumarin, heptaminol, and isometheteptene reversed the expression levels of HIGs and play a potential role in the treatment of CAC. Compared with the other three drugs not reported in CAC, metronidazole has been shown to inhibit the occurrence of tumors and reduce the degree of inflammation in CAC animal models [46]. Based on the ceRNA hypothesis, we predicted miRNAs and lncRNAs using HIGs and constructed a ceRNA-immunoregulatory network to better understand the CAC-related molecular mechanisms and biological phenomena at the transcriptional level.

To determine the underlying mechanisms affecting the occurrence of CAC, we analyzed the differences in pathways between the CAC and NC groups using two major pathway enrichment methods. The results showed that the largest number of genes (93 genes) was enriched in the NF-κB pathway, revealing that this pathway is closely related to CAC. The NF-κB pathway is involved in the immune response in vivo through classical and non-classical pathways, allowing the massive release of pro-inflammatory cytokines that cause tissue damage and participate in tumor invasion and metastasis by regulating the expression of angiogenesis-related genes [47–50]. A previous study showed that the NF-κB pathway exerts a tumor-promoting effect in a CAC mouse model [51].

We further evaluated alterations in the immune microenvironment of CAC and found that only neutrophils had the same expression trend (high in the CAC group and low in the NC group) across the three methods used to calculate the degree of immune cell infiltration. These results were consistent with those reported in previous studies showing that neutrophil infiltration is significantly higher in the colonic mucosal layer of a CAC mouse model, promoting CAC occurrence by secreting chemokines and, consequently, recruiting chemotactic receptors [52, 53]. Correlation analysis showed that the NF-κB pathway was positively correlated with neutrophils. Further analysis of 93 core genes enriched in the NF-κB pathway revealed that IL6ST was positively associated with neutrophils. We subsequently screened the miRNAs regulating IL6ST (miR-1-3p) and its upstream lncRNA from the ceRNA network (NEAT1). Previous studies suggested that NEAT1 promotes tumor development by downregulating target miRNAs. Zhou et al. [54] reported that NEAT1 targets and downregulates miR-500a-3p, promoting gastric cancer cell proliferation and invasion; Huang et al. [55] suggested that NEAT1 promotes pancreatic cancer progression by negatively regulating miR-506-3p; whereas Zhang et al. [56] showed that upregulation of NEAT1 is involved in the proliferation of glioma cells by negatively regulating miR-324-5p. However, the role of NEAT1 in CAC has rarely been reported. MiR-1-3p, a downstream target of NEAT1, has been shown to play a facilitative role in tumors. For instance, Peng et al. [57] reported that miR-1-3p affects gastric cancer cell proliferation by promoting the oxygen glycolytic pathway, and Liu et al. [58] indicated that downregulation of miR-1-3p expression is involved in the growth and motility of lung cancer cells. Our results suggest that IL6ST is a target gene of miR-1-3p in CAC. IL6ST is a subunit of the IL-6 receptor, and the IL-6/IL-6 receptor complex only functions by binding to IL6ST [59]. Previous studies showed that IL-6 promotes the occurrence of CAC by interacting with IL6ST [60]. Our data further supported that NEAT1 can competitively bind to miR-1-3p and upregulate IL6ST at the transcriptional level, affecting the NF-κB pathway and neutrophil infiltration as well as promoting the occurrence and development of CAC.

We constructed a ceRNA-immunoregulatory network by integrating multiple bioinformatics tools and deeply analyzed the alterations of the immune microenvironment and pathways in the process of CAC. Ultimately, we identified a ceRNA immunoregulatory axis closely related to CAC. However, our study had some limitations; (1) we only used public datasets for the analysis, which may be biased because of the limited sample size. (2) Although we conducted a bioinformatic analysis and database prediction, the direct relationship between the ceRNA immunoregulatory axes requires experimental verification. (3) In vivo and in vitro functional experiments are needed to determine the biological role of the ceRNA regulatory axis in CAC. (4) Currently, bioinformatic analysis for the prediction of drug utility is limited; however, we believe that this study may provide valuable insights in designing drugs to treat CAC.

## Conclusion

In conclusion, we identified a ceRNA immunoregulatory network of CAC and suggested that the NEAT1/miR-1-3p/IL6ST regulatory axis participates in the CAC process by altering neutrophil infiltration in the immune microenvironment. These findings provide a new perspective and direction for further exploration of the immunoregulatory mechanisms underlying CAC.

## Supplementary Information

**Additional file 1. Table S1**: Number of genes in the 10 modules. **Table S2**: List of 130 hub immune genes.

**Additional file 2. Fig. S1**: shows the stem-loop structure of miRNA precursors. (A) Secondary structures of human pre‐miR‐1. (B) Secondary structures of human pre‐miR‐133a.

## Data Availability

Publicly available datasets were analyzed in this study. These data can be found at https://www.ncbi.nlm.nih.gov/geo/.

## Abbreviations

CAC: Ulcerative colitis-associated colorectal cancer
ceRNA: Competing endogenous RNA
HIGs: Hub immune genes
UC: Ulcerative colitis
mRNA: Messenger RNA
miRNAs: Micro RNAs
IRGs: Immune-related genes
WGCNA: Weighted gene co-expression network analysis
NCBI: National Center for Biotechnology Information
GEO: Gene Expression Omnibus
NC: Normal control
RMA: Robust multiarray average
TOM: Topological overlap matrix
MEs: Module eigengenes
GS: Gene significance
GSEA: Gene set enrichment analysis
GSVA: Gene set variation analysis
MSigDB: Molecular Signatures Database
lncRNA: Long non-coding RNA
DEmRNAs: Differentially expressed mRNAs
DEmiRNAs: Differentially expressed miRNAs
PCA: Principal component analysis
ANOVA: After the analysis of variance
MM: Module membership
