# Supplementary material for: A novel ceRNA-immunoregulatory axis based on immune cell infiltration in ulcerative colitis-associated colorectal carcinoma by integrated weighted gene co-expression network analysis
Source: BMC Gastroenterol. 2022 Apr 15;22:188. doi: 10.1186/s12876-022-02252-7 (PMC9013140; doi:10.1186/s12876-022-02252-7)
Supplement: Supplementary file 1 — Additional file 1. Table S1: Number of genes in the 10 modules. Table S2: List of 130 hub immune genes. [file 12876_2022_2252_MOESM1_ESM.docx]

**Table S1** Number of genes in the 10 modules

| **Modules colors** | **Number** |
| --- | --- |
| black | 81 |
| blue | 1053 |
| brown | 388 |
| green | 130 |
| grey | 646 |
| magenta | 35 |
| pink | 42 |
| red | 98 |
| turquoise | 2264 |
| yellow | 243 |

**Table S2** List of 130 hub immune genes

| **Symbol** | **Type** | **Log2FC** | ***p*-value** | **Adj.*p*.val** |
| --- | --- | --- | --- | --- |
| ISG20 | up | 2.766639563 | 3.65E-09 | 1.58E-05 |
| FCGR2B | up | 2.542947131 | 2.04E-08 | 2.90E-05 |
| ICAM2 | up | 1.981626041 | 2.84E-08 | 3.33E-05 |
| CCL11 | up | 3.987037723 | 3.02E-08 | 3.34E-05 |
| PIK3R3 | up | 2.014139575 | 1.93E-07 | 7.65E-05 |
| CCR7 | up | 1.797816701 | 2.76E-07 | 9.18E-05 |
| S100A8 | up | 6.019661634 | 3.08E-07 | 9.32E-05 |
| SERPINA3 | up | 3.609869541 | 4.57E-07 | 0.000112495 |
| FGF9 | down | -1.456180462 | 4.81E-07 | 0.000116906 |
| MMP12 | up | 3.836718357 | 8.01E-07 | 0.000154902 |
| RAC2 | up | 2.01655173 | 9.51E-07 | 0.000165662 |
| CD40 | up | 1.872793028 | 9.77E-07 | 0.00016568 |
| CXCR4 | up | 3.219523032 | 9.93E-07 | 0.00016568 |
| S100P | up | 2.656325473 | 1.02E-06 | 0.00016568 |
| CKLF | up | 1.518913878 | 1.20E-06 | 0.000176012 |
| ITGB2 | up | 1.792336894 | 1.21E-06 | 0.000176012 |
| ADM | up | 1.547477907 | 1.36E-06 | 0.00019068 |
| FCER1G | up | 2.085701847 | 1.38E-06 | 0.00019068 |
| IFI30 | up | 1.225842716 | 2.10E-06 | 0.000245136 |
| GREM1 | up | 3.548200826 | 2.50E-06 | 0.000269261 |
| PRKCB | up | 2.401827662 | 2.77E-06 | 0.000283138 |
| CD247 | up | 1.127316089 | 2.93E-06 | 0.000289067 |
| AREG | up | 2.525708397 | 3.15E-06 | 0.000302179 |
| C3AR1 | up | 1.69581084 | 3.95E-06 | 0.000341008 |
| SDC2 | up | 1.758141754 | 6.17E-06 | 0.000448561 |
| CR2 | up | 3.406189874 | 7.14E-06 | 0.000486956 |
| GMFG | up | 1.537153001 | 8.27E-06 | 0.000529025 |
| CCL4 | up | 3.213267169 | 9.21E-06 | 0.000549433 |
| HCST | up | 1.026105389 | 9.88E-06 | 0.000570258 |
| LYN | up | 1.789745372 | 1.12E-05 | 0.000618688 |
| IL1RN | up | 3.334420644 | 1.41E-05 | 0.000716522 |
| IL1R1 | up | 1.0641696 | 1.43E-05 | 0.000718785 |
| CXCL1 | up | 3.663193622 | 1.45E-05 | 0.000728351 |
| MALT1 | up | 1.469404333 | 1.55E-05 | 0.000757286 |
| ECD | up | 1.058385318 | 1.80E-05 | 0.000831624 |
| CSF2RA | up | 1.28964971 | 1.85E-05 | 0.000839597 |
| REG1A | up | 5.861514866 | 2.03E-05 | 0.000893827 |
| DEFA5 | up | 6.309451441 | 2.10E-05 | 0.000910534 |
| TNC | up | 2.820473407 | 2.15E-05 | 0.000926745 |
| IDO1 | up | 3.650069011 | 2.24E-05 | 0.000951817 |
| PTGDS | up | 2.21087567 | 2.28E-05 | 0.000955957 |
| FCGR3B | up | 4.60454884 | 2.36E-05 | 0.000967689 |
| ANGPTL2 | up | 1.367186322 | 3.12E-05 | 0.001136774 |
| AKT3 | up | 1.297400659 | 3.64E-05 | 0.00125111 |
| DUOX2 | up | 3.788200881 | 3.71E-05 | 0.001271602 |
| APOBEC3G | up | 1.295055643 | 4.12E-05 | 0.001353651 |
| ITK | up | 1.499668566 | 4.32E-05 | 0.00138373 |
| CD3D | up | 1.208172403 | 4.77E-05 | 0.00147454 |
| PI3 | up | 2.513501106 | 4.79E-05 | 0.001478123 |
| S100A9 | up | 3.869436741 | 5.09E-05 | 0.001540746 |
| CCL21 | up | 2.118472455 | 5.55E-05 | 0.001631741 |
| CXCL9 | up | 3.12733716 | 5.81E-05 | 0.001699898 |
| CXCL13 | up | 3.466941728 | 6.07E-05 | 0.001749409 |
| IL1B | up | 4.284560477 | 7.15E-05 | 0.001953907 |
| TRBC1 | up | 1.410798767 | 7.27E-05 | 0.001972192 |
| CCR1 | up | 1.555728625 | 7.53E-05 | 0.002013069 |
| GZMB | up | 2.156820272 | 8.12E-05 | 0.002135923 |
| DMBT1 | up | 3.210696464 | 9.44E-05 | 0.002403427 |
| MMP9 | up | 2.398359148 | 0.000100878 | 0.002481849 |
| ROBO1 | up | 1.47026151 | 0.000112098 | 0.002627188 |
| CD79A | up | 1.785455197 | 0.000112271 | 0.002628137 |
| CD1C | up | 1.175910673 | 0.000119047 | 0.002719651 |
| IFNAR2 | up | 1.02668126 | 0.000126712 | 0.002858703 |
| CD4 | up | 1.063051619 | 0.000130161 | 0.002910134 |
| CCL18 | up | 2.029904238 | 0.000142599 | 0.003111418 |
| ICAM1 | up | 1.564592904 | 0.000148026 | 0.003191606 |
| CCL13 | up | 2.476588306 | 0.000148057 | 0.003191606 |
| IL10RA | up | 1.320289426 | 0.000177824 | 0.003648237 |
| CCL19 | up | 2.664641006 | 0.000179262 | 0.003670174 |
| LILRB3 | up | 1.10426275 | 0.000184412 | 0.00372962 |
| PF4 | up | 1.420281488 | 0.000202718 | 0.003966947 |
| C3 | up | 2.280218208 | 0.000216363 | 0.004123333 |
| CSF2RB | up | 1.735989707 | 0.000225996 | 0.004247223 |
| TLR2 | up | 1.847061081 | 0.000229131 | 0.004285938 |
| LCN2 | up | 2.144732611 | 0.000255418 | 0.004562706 |
| VCAM1 | up | 1.863705626 | 0.000287886 | 0.004927848 |
| IGF1 | up | 1.379130608 | 0.000303347 | 0.005083742 |
| IL15 | up | 1.420786791 | 0.00031611 | 0.005206499 |
| SEMA5A | down | -1.070911012 | 0.000351431 | 0.005609666 |
| RASGRP1 | up | 1.459397268 | 0.000363145 | 0.005741435 |
| TNFSF13B | up | 1.317144365 | 0.00036358 | 0.005743756 |
| IL13RA2 | up | 2.890311804 | 0.000439539 | 0.006529503 |
| DEFA6 | up | 4.206739567 | 0.000446155 | 0.006598256 |
| JAK1 | up | 1.038219407 | 0.000534255 | 0.007447795 |
| CXCL10 | up | 2.241308051 | 0.000556508 | 0.007639741 |
| HCK | up | 1.397548822 | 0.000558745 | 0.007644758 |
| MX2 | up | 1.046386778 | 0.000596571 | 0.007944045 |
| NPY | down | -1.192566692 | 0.000619841 | 0.0081504 |
| CTSE | up | 1.921393906 | 0.000634024 | 0.008265961 |
| NCK1 | up | 1.123404233 | 0.000775085 | 0.00947132 |
| PLAUR | up | 1.843652552 | 0.000972469 | 0.011085972 |
| LCP2 | up | 1.866084661 | 0.001216598 | 0.012856564 |
| CTSG | up | 1.580176381 | 0.001250774 | 0.01309336 |
| FGR | up | 1.357689014 | 0.001289284 | 0.013363069 |
| BID | up | 1.09985709 | 0.001421052 | 0.014268537 |
| HSP90AB1 | up | 1.17709074 | 0.001428215 | 0.014318806 |
| VIP | up | 2.22093341 | 0.001451362 | 0.014500273 |
| C5AR1 | up | 1.453726525 | 0.001476249 | 0.014682152 |
| LTF | up | 2.002329011 | 0.001752397 | 0.016552877 |
| APOD | up | 1.735661348 | 0.002110142 | 0.018977944 |
| CCL2 | up | 1.977426648 | 0.002443561 | 0.020834469 |
| CXCL3 | up | 1.809277409 | 0.002446662 | 0.020846854 |
| TNFAIP3 | up | 1.336268465 | 0.002768315 | 0.022646689 |
| JAK2 | up | 1.096250481 | 0.002897207 | 0.023338154 |
| CD8A | up | 1.245569736 | 0.003002365 | 0.023857244 |
| CSF3R | up | 1.515226948 | 0.0030652 | 0.024221281 |
| CD19 | up | 1.050572743 | 0.004601169 | 0.031595959 |
| OASL | up | 1.11701868 | 0.004690801 | 0.031994854 |
| GHR | down | -1.205349868 | 0.004853345 | 0.032696477 |
| S100A12 | up | 2.511977774 | 0.005051097 | 0.033596961 |
| IL18RAP | up | 1.191634637 | 0.005394675 | 0.035037592 |
| CXCL6 | up | 2.031055616 | 0.007025672 | 0.042072659 |
| FABP4 | up | 1.977518381 | 0.007475388 | 0.043837857 |
| MAP3K8 | up | 1.116874424 | 0.007588866 | 0.04425199 |
| LANCL1 | up | 1.001330877 | 0.007865556 | 0.045142535 |
| PLAU | up | 1.173082847 | 0.00809995 | 0.045984331 |
| PDK1 | up | 1.020458537 | 0.008492769 | 0.047543082 |
| AQP9 | up | 2.493874852 | 0.009285313 | 0.050580456 |
| HLA-DQB1 | up | 1.513030954 | 0.010554852 | 0.055187195 |
| IL6ST | up | 1.0269821 | 0.011569296 | 0.058623265 |
| WNT5A | up | 1.372121098 | 0.014863512 | 0.069165155 |
| TLR8 | up | 1.022317712 | 0.015753028 | 0.072091908 |
| TGFBR2 | up | 1.028113443 | 0.022317087 | 0.091391454 |
| PTGS2 | up | 1.568305786 | 0.023035477 | 0.093155395 |
| TLR4 | up | 1.086149565 | 0.023262844 | 0.093714684 |
| NR4A1 | up | 1.11494218 | 0.024293077 | 0.096665277 |
| IL1RL1 | up | 1.329628591 | 0.02594409 | 0.100492361 |
| CMTM2 | up | 1.011571175 | 0.032562458 | 0.116896317 |
| PROK2 | up | 2.214893641 | 0.033252805 | 0.118481333 |
| FPR2 | up | 1.352549752 | 0.037792401 | 0.128839665 |
