# Supplementary figures and images for: A novel ceRNA-immunoregulatory axis based on immune cell infiltration in ulcerative colitis-associated colorectal carcinoma by integrated weighted gene co-expression network analysis
Source: BMC Gastroenterol. 2022 Apr 15;22:188. doi: 10.1186/s12876-022-02252-7 (PMC9013140; doi:10.1186/s12876-022-02252-7)

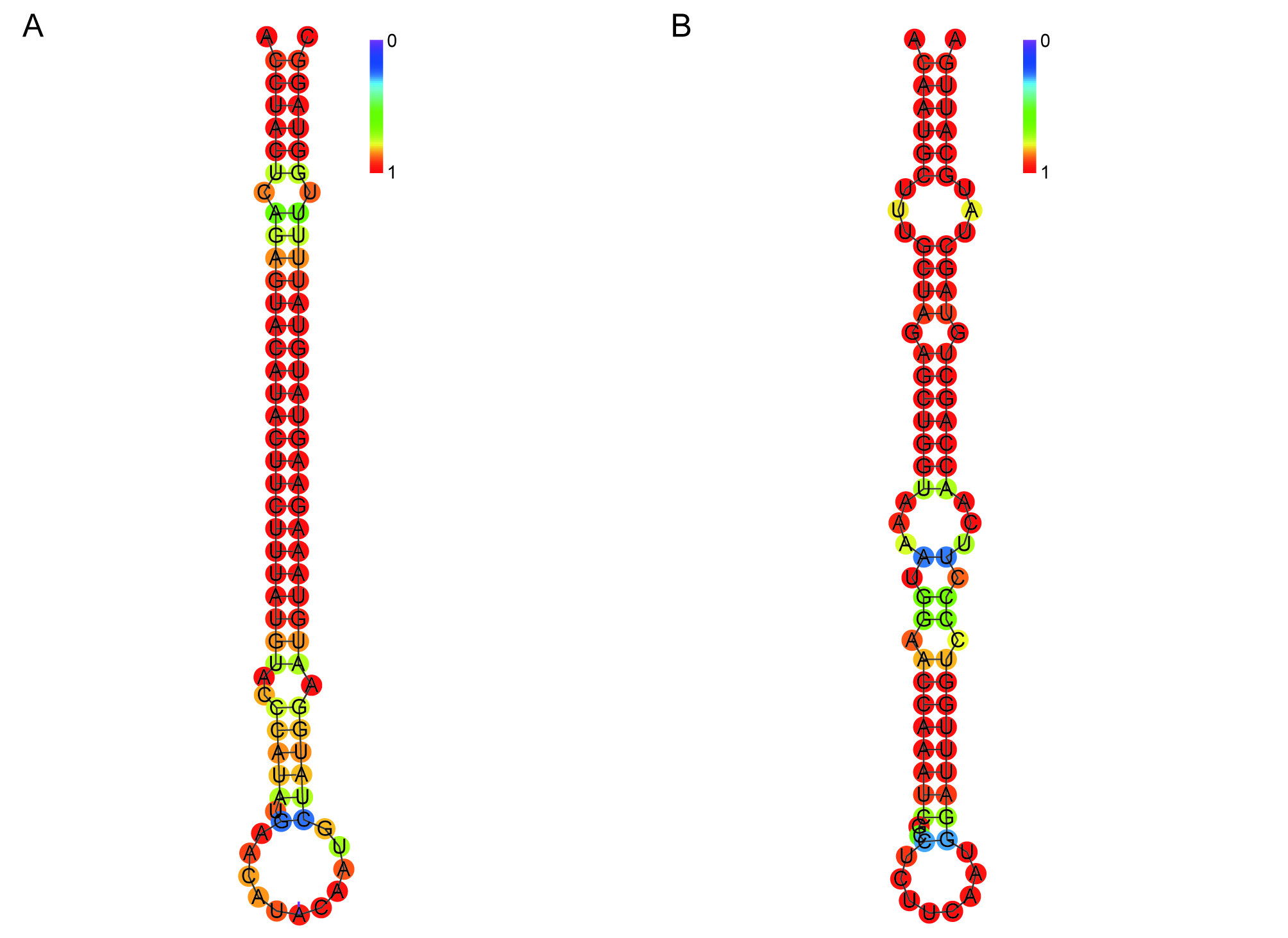

Supplement: Supplementary file 2 — Additional file 2. Fig. S1: shows the stem-loop structure of miRNA precursors. (A) Secondary structures of human pre‐miR‐1. (B) Secondary structures of human pre‐miR‐133a. [file 12876_2022_2252_MOESM2_ESM.tif]
